# Comparison of HDL-Associated Antioxidant Activities and Anti-Inflammatory Effect Between Ozonated Sunflower Oil (OSO) and Ozonated Olive Oil (OOO) Under Carboxymethyllysine-Induced Acute Phase in Zebrafish Adults and Embryos

**DOI:** 10.3390/antiox15070840

**Published:** 2026-07-03

**Authors:** Kyung-Hyun Cho, Krismala Djayanti, Ashutosh Bahuguna, Yunki Lee, Sang Hyuk Lee, Seung Hee Baek

**Affiliations:** Raydel HDL Research Institute, Medical Innovation Complex, Daegu 41061, Republic of Korea

**Keywords:** dyslipidemia, e-nose, fatty liver, inflammation, oxidative stress, ozonated oil, paraoxonase, senescence

## Abstract

This study compares the efficacy of ozonated sunflower oil (OSO) and ozonated olive oil (OOO) in terms of antioxidant properties, modulation of high-density lipoprotein (HDL) functionality, and protective effects against carboxymethyllysine (CML)-mediated stress in zebrafish embryos and adults. The spectral and electronic nose (e-nose) analyses revealed that OSO and OOO possessed markedly distinct physicochemical characteristics and volatile and olfactory constituents compared with non-ozonated sunflower (SO) and olive oil (OO). The fluorescence spectrum analysis of HDL treated with OOO and OSO exhibited a red shift (2.6~3.3 nm) in the wavelength maximum fluorescence (WMF), accompanied by pronounced quenching of tryptophan fluorescence. Additionally, a significant increase in HDL-associated paraoxonase (PON) and ferric ion reduction (FRA) activity was observed in the OSO- and OOO-treated HDL. However, compared to OOO, significantly higher PON and FRA activities were observed in HDL treated with OSO. Also, compared to OOO, OSO effectively reverses CML-induced oxidative stress, altered heart rate, and reduced embryo survival. Similarly, in adult zebrafish, CML-compromised survival, swimming impairment, and disturbed antioxidant parameters were prevented by treatment with OOO and OSO. Nonetheless, OSO showed significantly higher efficacy than OOO. Consistently, OSO substantially reduced the CML-elevated blood glucose, total cholesterol (TC), triglycerides (TG), and low-density lipoprotein cholesterol (LDL-C) levels with a marked increase in high-density lipoprotein cholesterol (HDL-C) levels. Notably, no significant effect of OOO was observed on the reduction in and augmentation of LDL-C and HDL-C, respectively. Both OOO and OSO significantly protect against CML-triggered liver and kidney damage. However, compared with OOO, OSO significantly reduced neutrophil infiltration, interleukin-6 (IL-6) production, liver steatosis, ROS generation, and cellular senescence in the kidneys. The study concludes that OSO exerts significantly higher beneficial effects than OOO on HDL functionality and antioxidant defense, thereby attenuating CML-induced inflammatory and oxidative damage.

## 1. Introduction

Ozone is a blue-colored gas with proven therapeutic effects against a variety of inflammatory disorders [1,2]. In particular, ozone’s established effect as a strong germicidal agent against a variety of bacteria, fungi, and viruses is well known [3]. The effect of ozone on the inhibition of nuclear factor-kappa B (NF-κB) has been documented to substantially prevent inflammation by inhibiting the expression of inflammatory mediators such as tumor necrosis factor-alpha (TNF-α), interleukin 6 (IL-6), IL-8, and interferon gamma (IFN-γ) [4]. Also, studies confirm the effect of ozone on nuclear factor erythroid 2-related factor 2 (Nrf2) activation [5,6], which positively impacts the generation of endogenous antioxidants such as superoxide dismutase (SOD), heme-oxygenase 1 (HO-1), reduced glutathione (GSH), and catalase (Cat). Ozone therapy was found to be effective in counteracting elevated total cholesterol (TC) and triglycerides (TG) in individuals with cardiopathy [7,8], hypertension, and ischemic disease [8]. Consistently, in psoriatic patients, ozone treatment showed substantial effects in mitigating inflammation, reducing TC and TG levels, and elevating high-density lipoprotein cholesterol (HDL-C).

Despite the several benefits of ozone, its short shelf life [9] remains a concern for its wide applicability. Fortunately, this can be improved manyfold by ozonation of herbal oils [10], such as sunflower oil (SO), olive oil (OO), and almond oil. During ozonation, ozone is trapped at unsaturated sites in oil components via the Criegee mechanism, forming distinct ozonated compounds such as peroxides and ozonides [11,12]. As ozone is primarily trapped in the unsaturated components of oils, oil composition also substantially affects the functionality of ozonated oil.

Among the different ozonated oils, ozonated olive oil (OOO) and sunflower oil (OSO) are commercially available and marketed for diverse health-promoting properties, primarily linked to antimicrobial and antioxidant activities [13]. Numerous reports have documented several health-beneficial effects of OSO. In the gastric mucosa of rats, OSO proved effective in augmenting intrinsic antioxidant levels and reducing TBARS (thiobarbituric acid reactive substances) [14]. In addition, OSO has been shown to treat atopic dermatitis by regulating the expression of filaggrin and thymic stromal lymphopoietin (TSLP) and by modulating mast cell infiltration [15]. In one of our earlier studies, OSO was shown to induce a red shift in the wavelength maximum fluorescence (WMF) accompanied by attenuation of HDL fluorescence intensity, indicating increased exposure of tryptophan residues to the aqueous polar environment [16]. Additionally, OSO exhibited a substantially more positive effect than SO in enhancing HDL-associated paraoxonase (PON) activity [16]. Likewise, studies have documented the beneficial effects of ozonated olive oil on glycemic control, systemic inflammation, and wound healing in patients with diabetic foot ulcers [17]. Also, OOO has been shown to ameliorate fatty liver, reduce TG, and exert an anti-inflammatory effect in diabetic and obese rodent models [18,19]. Numerous reports have documented the diverse biological functions of OSO [14,15,16] and OOO [17,18,19]; however, comparative investigations between these two remain limited, particularly regarding their impact on HDL function. In view of this, the present study aimed to compare the effects of OOO and OSO, and their parent oils (OO and SO), on in vitro antioxidant activity, physicochemical differences in HDL, and protective effects against CML-induced stress in zebrafish embryos. Furthermore, electronic nose (e-nose) analysis was performed to compare the volatile and olfactory characteristics of ozonated and non-ozonated oils. In addition, the comparative protective efficacy of OSO and OOO against the CML-induced mortality, oxidative stress, dyslipidemia, and hepatic and renal toxicity was investigated in adult zebrafish.

Zebrafish were chosen as a model organism due to their high genetic and physiological similarities to humans [20]. Lipid metabolism in zebrafish and humans shares many receptors, proteins, and enzymes. For instance, like humans, zebrafish express cholesteryl ester transfer protein (CETP), an important protein that facilitates cholesterol transfer between lipoproteins [21,22]. Unlike zebrafish, mice and rats lack CETP proteins [21]. Among the frequently used animal models, zebrafish are among the smallest (<5 cm) and have well-developed adaptive and innate immune systems [23]. Due to their small size and ease of maintenance, large numbers of zebrafish can be housed in small tanks. Based on these qualities, zebrafish are gaining significant attention as a preclinical model organism for studying various human diseases [24]; therefore, outcomes from zebrafish can provide valuable information for designing human studies.

## 2. Materials and Methods

### 2.1. Materials

Ozonated sunflower oil (OSO, Bodyone, Flambo oil) was provided complementarily by the National Center for Scientific Research (CNIC), Havana, Cuba. The sunflower oil (SO) was purchased from Ondoliva, Tudela, Spain. The used OSO had a peroxide value of 746.2 mmol-equiv/kg, an acidity of 2.45 mg KOH/g, an aldehyde concentration of 0.24 mmol/g, and a viscosity of 110.35 mPa.s and was free of pathogenic microbes. A detailed specification of the OSO is provided as Supplementary Table S1. The ozonated olive oil (OOO, Joy Love) was procured from the Wuhan Taiyang Biotechnology Co., Ltd. (Wuhan, China). The olive oil (OO, extra virgin) was purchased from Sajo Haepyo Co. (Seoul, Republic of Korea) (the oil was sourced from Spain). All other chemicals and reagents, unless otherwise stated, were of analytical grade and used as supplied.

### 2.2. Determination of pH and Ultraviolet (UV) Spectrum of Ozonated and Non-Ozonated Oil Samples

The pH and absorbance maximum wavelength (λ_max_) of ozonated (OOO, OSO) and non-ozonated (SO, OO) oils were determined using the digital pH meter (Orion Star A211, Thermo-Fisher Scientific, Chelmsford, MA, USA) and UV–visible spectrophotometer (UV 2600i, Shimadzu, Kyoto, Japan). Each 0.5% (final, *v*/*v*) solutions of SO, OO, OSO, and OOO were used to determine the pH. Likewise, a 0.5% solution was scanned at wavelengths of 150 to 350 nm to obtain the absorbance spectrum.

### 2.3. Electronic Nose Analysis of Ozonated and Non-Ozonated Oil Samples

For the electronic nose (e-nose) analysis, a Heracles II ultra-fast gas chromatography (Alpha MOS, Toulouse, France)-based electronic nose equipped with dual capillary columns (MXT-5 and MXT-1701) (Restek, Bellefonte, PA, USA) was used. For each oil sample (SO, OO, OSO, and OOO), 1 g of the sample (*n* = 5) was transferred into a 20 mL glass vial and sealed with a magnetic spectrum cap. Headspace generation was performed using an autosampler by incubating the vials at 40 °C for 20 min. Subsequently, a 1000 μL aliquot of headspace gas was injected into the system at a flow rate of 125 μL/sec, with the injector temperature maintained at 200 °C. The column temperature program was set as follows: initially held at 40 °C for 5 s, increased to 100 °C at a rate of 0.5 °C/sec, and then raised to 250 °C at 20 °C/sec, followed by a final hold for 20 s, resulting in a total acquisition of 220 s. The separated volatile components were detected using a flame ionization detector (FID) maintained at 260 °C. Data processing, multivariate analysis, and Kovats retention index calculations were performed using the AlphaSoft platform, version 14.2 (Alpha MOS, Toulouse, France), based on a C6-C16 n-alkane standard mixture.

### 2.4. In Vitro Antioxidant Activity

The antioxidant activity was determined using the diphenyl-1-picryhydrazyl (DPPH) free radical scavenging activity and ferric ion reduction ability (FRA), as in a previously described method [16]. Briefly, 20 μL (final 5%) of SO, OO, OSO, and OOO was mixed with 180 μL of DPPH solution (prepared by dissolving 0.24 mg DPPH in 10 mL methanol). The absorbance at 517 nm was recorded at different time points (0, 2, 4, 6, and 24 h) using a spectrophotometer.

For the determination of FRA, 20 μL (final 5%) of SO, OO, OSO, and OOO was mixed with 180 μL of freshly prepared FRA reagent (prepared by blending 10:1:1 (*v*/*v*) of 0.3 M sodium acetate, 0.01 M 2,4,6-tripridyl-*S* triazin, and 0.02 M ferric chloride). Finally, the absorbance at 593 nm was recorded at different time points (0, 2, 4, 6, and 24 h) using a spectrophotometer.

### 2.5. Purification of High-Density Lipoprotein (HDL) from Human Blood

The HDL was isolated from human blood, obtained from three voluntary participants (25 ± 3 years). Informed consent was obtained from the participants donating blood for the study. Blood was collected after 12 h of fasting, in accordance with the Helsinki guidelines, as adopted by the Institutional Board of the Korea National Institute for Bioethics Policy (KoNIBP, approval no. P01-202109-31-009, date of approval 27 September 2021). Blood was centrifuged to obtain serum, which was then processed for sequential density-gradient ultracentrifugation. The density was adjusted using NaCl and NaBr [25]. In brief, 5 mL of serum was adjusted to a density of 1.019 < d < 1.063 g/mL and centrifuged for 24 h at 100,000× *g* to remove the very-low-density lipoprotein (VLDL) and low-density lipoprotein (LDL) fractions. The remaining solution density was adjusted to 1.063 < d < 1.125 g/mL. After 24 h of centrifugation at 100,000× *g*, the HDL fraction was collected and dialyzed overnight using Tris-buffered saline (TBS, pH 8.0). Afterward, the dialyzed HDL was preserved in the refrigerator for further use.

### 2.6. Wavelength Maximum Fluorescence of HDL

Individually, 40 μL of HDL (1 mg/mL in TBS) was mixed with 360 μL of SO, OO, OSO, or OOO dissolved in TBS (final 1%). A time-dependent (0–48 h) wavelength maximum fluorescence (WMF) of the tryptophan residue was examined at the excitation wavelength of 295 nm and the emission spectrum at 305–400 nm [16].

### 2.7. Effect of Ozonated Oils on HDL-Associated Paraoxonase and Ferric Ion Reduction Activity

The effect of ozonated (OOO, OSO) and non-ozonated (OO, SO) oils on the paraoxonase (PON) and FRA of HDL was determined using the earlier described method [26]. In brief, 20 μL of HDL (1 mg/mL) was mixed with 170 μL of 1% SO/OO/OSO or OOO dissolved buffer [Tris-HCl (90 mM), NaCl (3.6 mM), CaCl_2_ (90 mM)]. Subsequently, 10 μL of 0.7 M paraoxon-ethyl was added, and the mixture was incubated for 60 min at 25 °C. Finally, absorbance at 415 nm was recorded, and the PON activity was determined using the molar absorbance coefficient (1.7 × 10^4^ M^−1^cm^−1^) of the *p*-nitrophenol (product formed).

For the FRA activity, 20 μL of HDL (1 mg/mL) was mixed with 20 μL of SO/OO/OSO or OOO (final 1%) and 160 μL of freshly prepared FRA reagent [0.3 M sodium acetate, 0.01 M 2,4,6-tripridyl-*S* triazin, and 0.02 M ferric chloride mixed in 10:1:1 (*v/v*)]. Finally, the absorbance at 593 nm was recorded after 60 min of incubation.

### 2.8. Zebrafish Rearing

A wild-type AB strain of zebrafish (14 weeks old) was reared in the glass tank equipped with a circulating water supply (28 °C) under an alternative photoperiod of 14 h light and 10 h dark. The zebrafish were maintained in accordance with the Animal Care and Use guidelines adopted by the Raydel Research Institute (RRI-24-001, approval date 2 September 2024). The used water was free of pathogenic coliforms and had a total bacterial load of <100 colony-forming units (cfu), a turbidity of 1.6 nephelometric turbidity units (NTU), residual chlorine of 0.18 mg/mL, and a pH of 7.3. The water was certified as safe for animal and human use following the water analysis done by Kirim Life Science Co., Ltd., Daegu, Korea (Supplementary Table S2). Twice (9 am and 6 pm) per day, commercial fish food (Tetrabit GmbH, D49307, Melle, Germany) was supplied to zebrafish at a rate of 20 mg/fish/day. Zebrafish were acclimated to these conditions for one week prior to further experiments.

### 2.9. Embryo Production and Treatment

To produce embryos, two female and one male zebrafish were kept in the breeding tank. Male and female zebrafish were separated overnight using a physical divider. In the morning, the divider was removed, allowing the male and female zebrafish to mate undisturbed for 30 min. After that, the produced embryos were collected and kept in 0.01% (*w/v*) seawater containing 0.1 mg/mL methylene blue.

The collected embryos were randomly divided into six groups (*n* = 150/group). Zebrafish in group I received a 10 nL microinjection of phosphate-buffered saline (PBS). Zebrafish in group II were microinjected with 10 nL PBS, containing 500 ng of CML. Embryos in groups III and IV were microinjected with 10 nL of PBS containing CML (500 ng), OO (final 1%), and SO (final 1%), respectively. Embryos in groups V and VI were injected with 10 nL of PBS containing CML (500 ng), OOO (final 1%), and OSO (final 1%). The selected CML concentration was based on a previous study [27] that reported severe toxicity in zebrafish embryos following CML exposure. The microinjection was performed under the microscope using a glass microcapillary needle in a pneumatic pump (PV803; World Precision Instruments, Sarasota, FL, USA) equipped with a magnetic manipulator (MM33, Kantech, Bensenville, IL, USA). The survivability, developmental deformities, and heartbeats of zebrafish across groups were monitored under the microscope.

### 2.10. Dihydroethidium (DHE) and Acridine Orange (AO) Staining

Embryos (*n* = 10/group) were transferred into a 24-well culture plate, washed twice with water, then treated with a 0.5 mL solution containing dihydroethidium (DHE, 30 μM) and acridine orange (AO, 5 μg/mL). After 30 min of incubation in the dark at room temperature (RT), embryos were washed with water (twice) and visualized under a fluorescence microscope at excitation and emission wavelengths of 505/535 nm and 585–615 nm for AO and DHE fluorescence detection, respectively.

### 2.11. Acute Toxicity in Adult Zebrafish

Zebrafish (*n* = 160) were randomly allocated into four groups (*n* = 40/group). In the PBS group, zebrafish were intraperitoneally injected with 10 μL of PBS, while in the CML group, zebrafish were intraperitoneally injected with 10 μL of PBS containing CML (final concentration: 3 mM). The selected CML concentration was based on the previous study [27], in which CML exposure induced severe acute toxicity and organ injury in adult zebrafish. The zebrafish in the OOO and OSO groups received a 10 μL microinjection of PBS containing CML (final 3 mM) and OOO (final 1%) or OSO (final 1%), respectively. All the fish from the specified groups (*n* = 40) were randomly subdivided into four tanks (i.e., *n* = 10/tank).

Zebrafish swimming ability [28] and survivability were assessed during 90 min post-treatment following the OECD 2019 guidelines [29].

### 2.12. Collection of Blood and Organs

After 90 min of treatment, zebrafish from all groups were euthanized by hypothermic shock. Immediately, blood was collected from the heart using a 22-G needle. Blood was collected separately from four tanks (*n* = 4) of the specified group (as mentioned in Section 2.11). From the single tank, zebrafish blood was collected, pooled in a single tube, and mixed with 1 mM ethylenediaminetetraacetic acid (EDTA) at a 2:3 ratio. The blood was centrifuged at 6000 rpm (10 min), and the supernatant was collected and stored in the refrigerator for further analysis.

Livers and kidneys from zebrafish were excised under a stereomicroscope and stored in 10% formalin for further histological and immunohistochemical analysis.

The 90 min time point for blood and organ collection was selected based on previous studies showing that CML exposure induces marked changes in blood biochemical parameters and histology within 60 min of treatment [27]. Furthermore, CML has been shown to induce sepsis-like hyperinflammatory responses [30], leading to rapid changes in blood biochemical parameters and organ damage, indicating that early time points are appropriate for assessing pathological changes.

### 2.13. Blood Biochemical Analysis

Plasma obtained from the different groups was processed to quantify total cholesterol (TC), triglycerides (TG), high-density lipoprotein cholesterol (HDL-C), and aspartate and alanine aminotransferase (AST and ALT) according to the manufacturers’ standard protocols. A detailed methodology is provided in Supplementary Method Section S1.

Plasma malondialdehyde (MDA), sulfhydryl content, PON-like activity, and FRA activity were quantified following the previously described method. A detailed procedure is provided in Supplementary Method Section S2.

### 2.14. Histological Examination

The tissue section was embedded in the FSC 22 frozen solution (Leica, Nussloch, Germany) and immediately placed in the deep refrigerator (–21 °C) overnight. The frozen tissue-embedded block was processed for cryo-sectioning using a Leica CM-1510S cryo-microtome (Nussloch, Germany). The 7 μM thick section of liver and kidney was processed for the hematoxylin and eosin (H&E) staining [31] to examine the morphological changes.

Lipid accumulation in the liver was determined by Oil Red O (ORO) staining. The liver tissue section (7 μM thick) was covered with ORO solution and incubated at 60 °C. After 5 min of incubation, the stained section was washed with 60% isopropanol, air-dried, and visualized under a microscope.

The senescence in the tissue section was determined by senescent-associated β-galactosidase (SA-β-gal) staining. The tissue section was stained for 16 h using 0.5 mL of 5-bromo-4-choloro-3-indolyl-β-D-galactopyranoside (X-Gal, 0.1%) solution in a moist environment. Finally, the stained section was washed three times with water and examined under the microscope for blue-stained cells.

### 2.15. Immunohistochemical (IHC) Analysis, Dihydroethidium (DHE), and Acridine Orange (AO) Staining

Interleukin (IL)-6 in the hepatic section was determined using the immunohistochemical (IHC) analysis. The tissue section was incubated for 16 h with anti-IL-6 primary antibody (200× diluted, ab9324, Abcam, Cambridge, UK) in a cool (4 °C) and moist environment. Afterward, the section was developed using a horseradish peroxidase (HRP)-linked secondary antibody (1000× diluted) as part of the EnVision HRP-polymer kit (K4001, Dako, Glostrup, Denmark) and a chromogenic substrate. Finally, the section was visualized under the microscope.

The DHE and AO fluorescent staining of tissue sections was performed as described in Section 2.10.

### 2.16. Statistical Analysis

All experiments were carried out at least in triplicate (*n* = 3), including in vitro, blood biochemical, histological, and electronic nose analyses, and the results are presented as the mean value ± standard error of the mean (SEM). The statistical difference among the multiple groups was assessed using one-way analysis of variance (ANOVA), followed by Tukey’s post hoc test, in the Statistical Package for the Social Sciences (SPSS, version 29; Chicago, IL). The pairwise comparison between the groups was determined by a *t*-test. The normality of the data was checked prior to performing parametric tests.

## 3. Results

### 3.1. Properties of Oils and UV–Visible Spectrum Analysis

A slightly higher density of OOO (0.913 mg/mL) than that of non-ozonated OO (0.911 mg/mL) was observed. Similarly, the density of SO (0.913 g/mL) enhanced slightly after ozonation (OSO, 0.915 g/mL). The pH of SO was acidic (Table 1) and became slightly more acidic following ozonation. Likewise, the OO acidic pH decreased after ozonation (Table 1). This study underscores that ozonation slightly enhances the density and reduces the pH of vegetable oils.

Spectral analysis of OO showed a relatively low absorbance intensity with shorter wavelength maxima (208 nm) (Supplementary Figure S1). Following ozonation, of OO (i.e., OOO) showed a bathochromic (red) shift of ~28 nm with an increase in absorbance intensity compared to the OO, suggesting the formation of strong absorbing chromophores. In contrast, the SO showed a higher wavelength maximum (232 nm), with a hypsochromic (blue) shift of ~16 nm and relatively low absorbance intensity following ozonation (i.e., OSO). The UV spectroscopic analysis revealed spectral changes in OO and SO after ozonation, suggesting the formation of new species that alter their optical properties.

### 3.2. Electronic Nose Analysis to Detect the Volatile Profile of Oil Samples

The electronic nose (e-nose) analysis revealed distinct profiles of volatile compounds, including aldehydes, alcohols, hydrocarbons, sulfur-containing compounds, and furans, demonstrating clear differences in aroma profile among the samples, as depicted in Table 2. Notably, hexanal exhibited the highest relative intensity in OSO and OOO following ozonation, indicating lipid peroxidation induced by ozonation. Hexanal is known to contribute green, grassy, leafy, and fatty sensory characteristics. Similarly, the abundance of the hydrocarbons hexane and heptane increased after ozonation. These compounds are associated with alkane-like fruity and sweet aromas and may originate from lipid degradation products. In addition, the elevated levels of pentan-2-ol and the exclusive detection of 3-pentanol in ozonated samples (OSO and OOO), compared with native oils, contributed to the distinct aroma characteristics of these groups.

**Figure 1:**
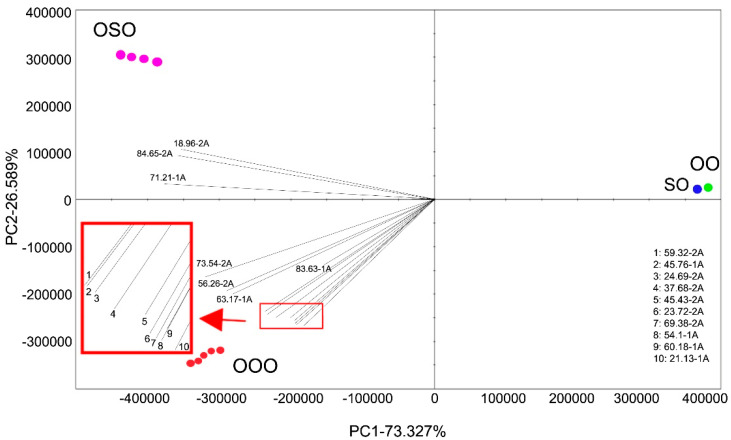
Principal component analysis (PCA) biplot, generated based on the volatile compounds detected in sunflower oil (SO), olive oil (OO), ozonated sunflower oil (OSO), and ozonated olive oil (OOO) samples using the electronic nose (e-nose). The numerical values marked with 1A and 2B (within the graph) represent the retention times of the compounds on the MXT-5 and MXT-1701 capillary columns, respectively. The PCA biplot was constructed from volatile profiles obtained from five replicates (*n* = 5) for each group.

Principal component analysis (PCA) showed that PC1 and PC2 together accounted for 99.9% of the total variance, with clear, distinct grouping patterns among the samples (Figure 1). SO and OO are positioned on the positive side if PC1 differs significantly from OSO and OOO. Compounds clustered in the negative regions of PC1 and PC2 were primarily associated with OOO, indicating a greater contribution of sulfur-containing and aldehyde compounds. In contrast, compounds grouped in the upper-left quadrant were associated with OSO and were characterized by relatively high aldehyde and hydrocarbon contents. Overall, the clustering pattern suggests that the oil sample’s aroma profiles are chemically distinct and can be effectively differentiated using the e-nose system.

### 3.3. Antioxidant Ability

**Figure 2:**
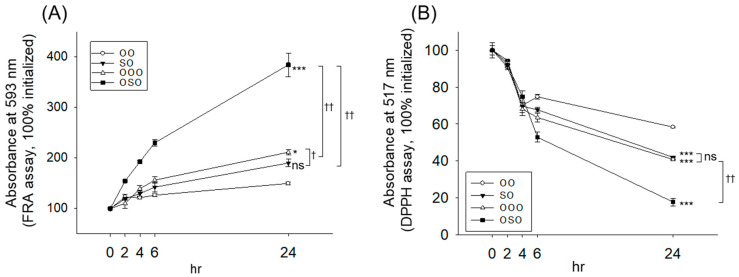
Comparison of in vitro (**A**) DPPH free radical scavenging activity and (**B**) ferric ion reduction (FRA) ability of olive oil (OO), sunflower oil (SO), ozonated olive oil (OOO), and ozonated sunflower oil. Each point represents the mean ± SEM from three (*n* = 3) independent experiments. * and *** highlight the statistical difference at *p* < 0.05 and *p* < 0.001, respectively, compared to OO, using one-way ANOVA following Tukey’s post hoc analysis. ^†^ (*p* < 0.05) and ^††^ (*p* < 0.01) represent the statistical difference between the marked groups evaluated by *t*-test. ns represents the non-significant difference.

FRA and DPPH free radical scavenging activity were assessed to determine the in vitro antioxidant activity of oils and their ozonated products. As depicted in Figure 2A, the highest FRA was observed for the OSO, which was, significantly, 1.8-fold higher than the FRA value detected for OOO at 24 h incubation. Also, compared to the non-ozonated SO, OSO displayed a significantly 2-fold higher FRA activity. A similar trend was observed for OOO, which showed notable 1.5-fold higher FRA activity than that of the OO. Interestingly, compared to OO, SO has higher FRA activity.

Similarly to the FRA, the DPPH assay showed the highest free radical scavenging ability for OSO, which was ~2.3-fold more than the radical scavenging activity of OOO and SO (Figure 2B). Compared with the OO, OOO has a significantly 1.4-fold higher DPPH radical-scavenging activity. The results showed that ozonation substantially augmented the antioxidant potential of SO and OO, as reflected by the higher antioxidant activity of OSO and OOO.

### 3.4. Fluorescent Intensity of HDL and HDL-Associated Antioxidant Ability

**Figure 3:**
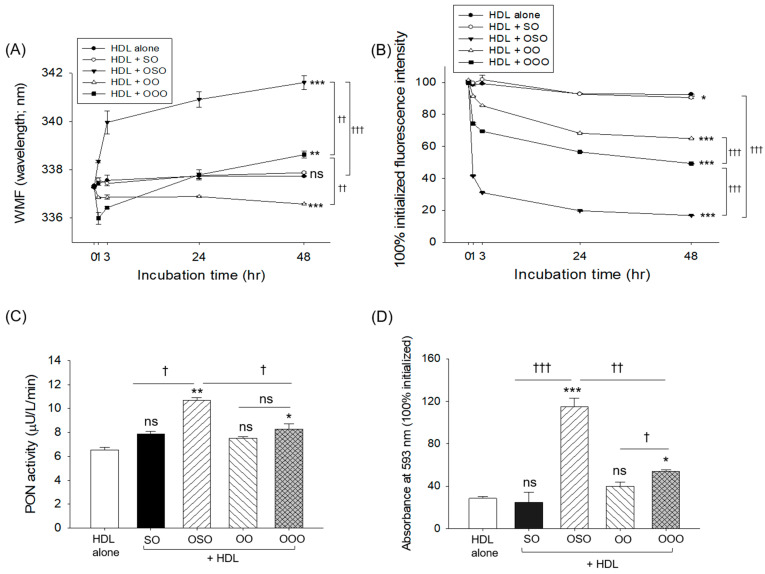
Comparative effects of sunflower oil (SO), olive oil (OO), ozonated sunflower oil (OSO), and ozonated olive oil (OOO) on high-density lipoprotein (HDL) fluorescence properties and antioxidant activities. (**A**) Wavelength maximum fluorescence (WMF) and (**B**) fluorescent intensity at WMF of HDL. (**C**) Paraoxonase (PON) and (**D**) ferric ion reduction activity (FRA) of HDL. Each data point represents the mean ± SEM from three independent experiments (*n* = 3). The symbols * (*p* < 0.05), ** (*p* < 0.01) and *** (*p* < 0.001) indicate statistical differences relative to the HDL-alone group, using one-way ANOVA following Tukey’s post hoc analysis. The symbols ^†^, ^††^ and ^†††^ underscore the statistical difference between the marked groups at *p* < 0.05, *p* < 0.01 and *p* < 0.001, evaluated by *t*-test. A non-significant difference is depicted by “ns”.

The fluorescence intensity results suggest a red shift in the WMF of HDL in response to OSO (~3.3 nm) and OOO (~2.6 nm) after 48 h of incubation (Figure 3A). However, compared to OOO, the WMF showed a greater degree of red shifting in response to OSO. Unlike the ozonated samples (OSO and OOO), the non-ozonated oils (SO and OO) showed little variation in WMF.

Furthermore, a severe quenching of WMF fluorescence (~83%) in HDL was observed in response to OSO after 48 h of incubation, compared with the initial fluorescence intensity (Figure 3B). Compared with SO, the OSO exhibited a notable, 4.9-fold lower fluorescence intensity after 48 h of incubation. OOO showed a lower degree of fluorescence quenching (~51%), which was merely 1.2-fold lower than the fluorescence intensity detected in OO. The findings suggest that ozonation has a substantial impact on the WMF shifting and intensity compared to the non-ozonized sample.

The PON activity of HDL was significantly 34% (*p* < 0.05) and 11% (*p* < 0.05) elevated by the exposure of OSO and OOO related to SO and OO, respectively (Figure 3C). However, compared to OOO, significantly 28% higher (*p* < 0.05) PON activity was noticed in the OSO-treated HDL, reflecting a higher impact of OSO than OOO on the augmentation of PON activity. No significant effect of SO and OO was observed on the modulation of PON activity compared with non-treated HDL.

Consistent with PON activity, significantly higher, by 4.9-fold (*p* < 0.001) and 3.1-fold (*p* < 0.01), FRA activity was observed in the OSO and OOO treated HDL than that of the SO and OO (Figure 3D). No significant effect of SO and OO was observed on FRA activity compared with untreated HDL. The collective findings highlight the impact of ozonated oils on enhancing HDL-associated PON and FRA activity.

### 3.5. Embryo Survivability in the Presence of CML

**Figure 4:**
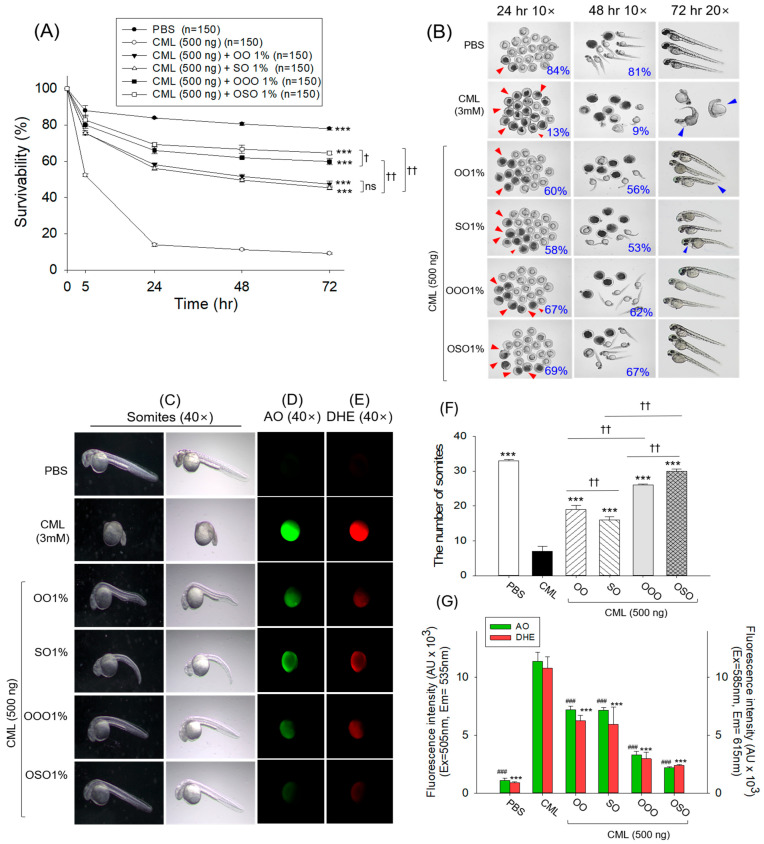
Comparative effects of olive oil (OO), sunflower oil (SO), ozonated olive oil (OOO), and ozonated sunflower oil (OSO) on carboxymethyllysine (CML)-induced toxicity in zebrafish embryos. (**A**) Survivability kinetics (0–72 h). Each data point represents the mean ± SEM from 150 embryos, divided into three groups (*n* = 3; 50 × 3 = 150). (**B**) Images of embryos post 24, 48, and 72 h treatment. The red arrow indicates dead embryos, while the blue arrow indicates developmental deformities in embryos. (**C**) Representative images depicting somites. (**D**,**E**) Dihydroethidium (DHE) and acridine orange (AO) staining, respectively. (**F**,**G**) Quantification of average somite counts and fluorescent intensity, respectively. The statistical difference *(p* < 0.001; *** for survaibility, somites counts and DHE fluorescence results, whereas *p*<0.001; ^###^ for AO fluorescence results ) was determined using one-way ANOVA with Tukey’s post hoc analysis compared to the CML-injected group. *p* < 0.05 (^†^) and *p* < 0.01 (^††^) represent the statistical difference between the marked groups determined by the *t*-test. A non-significant difference is depicted by “ns”.

Embryo survivability declined sharply after 5 min post-treatment of CML, which gradually decreased over time and finally reached 9.5% after 72 h post-treatment, which was, significantly, 8.4-fold lower than the survivability observed in the PBS (control) group (Figure 4A,B). Treatment of both SO and OO has a substantial protective effect on CML-impaired embryo survivability, as evidenced by ~4.8-fold higher survivability than that of the CML-treated group at 72 h post-treatment. Compared to SO and OO, OSO and OOO exhibit notably higher embryo survivability (1.4-fold and 1.2-fold, respectively), attesting to the impact of ozonation on the augmentation of the bio-functionality of vegetable oils. Notably, compared to the OOO, a significantly higher (*p* < 0.05) embryo-protective effect for OSO was observed, attesting to its functional benefit over OOO.

Also, a reduced average somite count (7) compared to the PBS control (33) was observed in the CML-injected groups, indicating CML’s adverse impact on embryonic development (Figure 4C,F). In contrast, the somite counts were substantially elevated to 19, 16, 26, and 30 following treatments with OO, SO, OOO, and OSO, respectively. In addition, DHE and AO fluorescent staining revealed a high prevalence of ROS and apoptosis in the CML-injected groups, which were ~10-fold higher than in the PBS (control) group (Figure 4D,E,G). Treatment with both OO and SO and their respective ozonated products, OOO and OSO, substantially reduced CML-elevated ROS generation and apoptosis. However, the efficacy of OOO and OSO was 2.1-fold and 2.5-fold higher in reducing ROS generation and 2.1-fold and 3.2-fold higher in reducing apoptosis, compared to OO and SO, respectively. Notably, treatment with 1% OO, SO, OOO, or OSO alone (without CML) did not adversely affect embryo survival, ROS production, apoptosis, and somite counts (Supplementary Figure S2). These parameters were comparable to those of the PBS (control) group, indicating that the treatments are non-toxic to embryos.

### 3.6. Heartbeat and Developmental Morphology of Embryos

**Figure 5:**
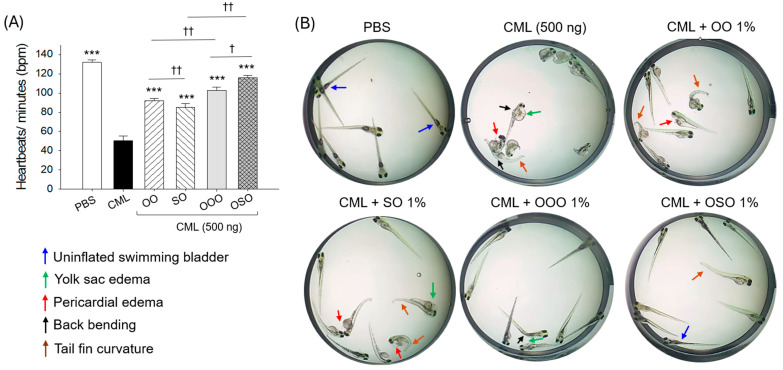
The comparative effects of olive oil (OO), sunflower oil (SO), ozonated olive oil (OOO), and ozonated sunflower oil (OSO) on CML-triggered adverse events in zebrafish embryos. (**A**) Heartbeat calculated at 72 h post-treatment. Each point represents the mean ± SEM from three (*n* = 5) independent experiments. (**B**) Pictorial view of the representative embryos at 144 h post-treatment. Blue arrows highlight the uninflated swimming bladder; green and red arrows represent yolk sac edema and pericardial edema, respectively, while brown and black arrows represent tail fin curvature and back bending, respectively. The statistical difference *p* < 0.001 (***) corresponds to the CML-injected group using one-way ANOVA following Tukey’s post hoc analysis. *p* < 0.05 (^†^) and *p* < 0.01 (^††^) represent the statistical difference between the marked groups determined by the *t*-test.

The normal average heartbeat was 132 beats per minute (bpm), observed in the PBS (control) group, which was significantly reduced to 51 bpm by treatment with CML (Figure 5A). The treatment of ozonated and non-ozonated vegetable oils significantly restored the CML-compromised heartbeat of zebrafish embryos. However, the OSO showed the greatest effect (116 bpm), followed by OOO (102 bpm), in improving the CML-impaired heartbeat.

The morphological data obtained at 144 h post-treatment suggest that 65% of the surviving embryos in the CML-treated group exhibited severe developmental defects, including stunted growth, tail fin curvature, back bending, yolk sac, and pericardial edema (Figure 5B). In contrast, only 34% and 40% of the embryos survived in CML co-treated with OO and SO, respectively, showing developmental deformities. At least (~15%) developmental deformities appeared in most of the surviving embryos from the CML co-treated with OOO and OSO. Combined results from embryo survivability and morphological analyses revealed a positive impact of ozonation on the functionality of OO and SO. Notably, the effect of the 1% OO, SO, OOO, and OSO treatments (without CML) on the heartbeat and embryo morphology was comparable to that of the PBS (control) group, indicating neither ozonated nor non-ozonated oils exerted a toxic effect (Supplementary Figure S3).

### 3.7. Anti-Inflammatory Activity in Adult Zebrafish

**Figure 6:**
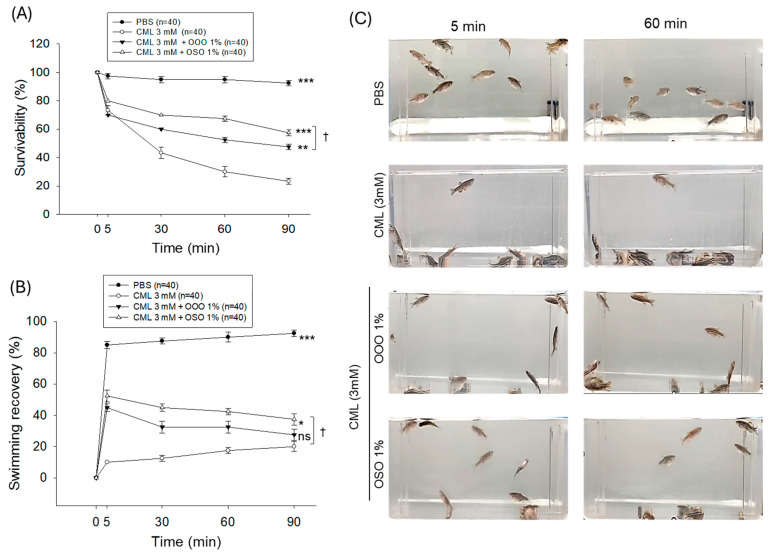
The comparative effects of olive oil (OO), sunflower oil (SO), ozonated olive oil (OOO), and ozonated sunflower oil (OSO) on the (**A**) survivability and (**B**) swimming activity of zebrafish impaired by exposure to carboxymethyllysine (CML). (**C**) Representative images depicting the swimming activity of zebrafish at 5 to 90 min post-treatment. Each point in the line graph represents the mean ± SEM value obtained from four (*n* = 4) independent experiments. The statistical differences *p* < 0.05 (*), *p* < 0.01 (**) and *p* < 0.001 (***) correspond to the CML-injected group using one-way ANOVA following Tukey’s post hoc analysis. *p* < 0.05 (^†^) represents the statistical difference between the marked groups determined by the *t*-test; a non-significant difference is depicted by “ns”.

As shown in Figure 6A, the adult zebrafish survivability declined rapidly with time in the CML-injected group. At 90 min post-treatment, 23.3% survivability was observed in the CML-injected group against the 92.5% survivability of the PBS (control) group. The treatment of OOO and OSO substantially improved the survivability of CML-compromised zebrafish, as shown by significantly higher survivability in the OOO- and OSO-supplemented groups than in the CML-injected group (2.1-fold and 2.5-fold, respectively). Compared with OOO, the OSO treatment showed 1.2-fold higher survivability, indicating greater efficacy of OSO over OOO.

Like the outcomes of survivability, the least swimming recovery of zebrafish was observed in the CML-injected group (Figure 6B,C). At 90 min post-injection, only 20% swimming recovery was observed in the CML-injected groups, which was significantly enhanced to 38% by OSO treatment. Interestingly, swimming recovery in the OOO-treated group showed a non-significant improvement (*p* > 0.05) compared with the CML-injected group.

### 3.8. Plasma Lipid Profile and Glucose Level

**Figure 7:**
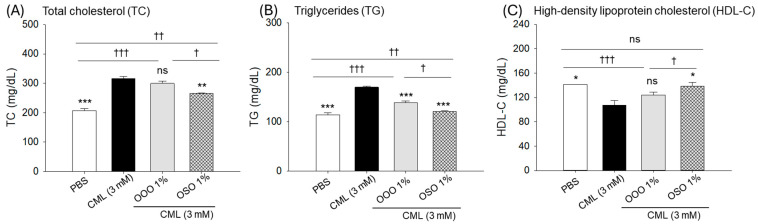

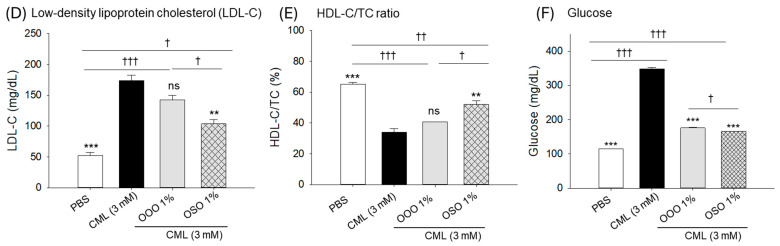
Comparative effects of olive oil (OO), sunflower oil (SO), ozonated olive oil (OOO), and ozonated sunflower oil (OSO) on the plasma lipid profile (**A**–**E**) and blood glucose (**F**) levels of carboxymethyllysine (CML)-treated zebrafish. Each point in the bar graph represents the mean ± SEM value obtained from four (*n* = 4) independent experiments. The statistical differences *p* < 0.05 (*), *p* < 0.01 (**) and *p* < 0.001 (***) correspond to the CML-injected group using one-way ANOVA following Tukey’s post hoc analysis. *p* < 0.05 (^†^), *p* < 0.01 (^††^) and *p* < 0.001 (^†††^) represent the statistical difference between the marked groups determined by the *t*-test; non-significant difference is depicted by “ns”.

The basal levels of TC (207.3 ± 6.3 mg/dL), TG (114.1 ± 4.6 mg/dL), and LDL-C (52.4 ± 4.9 mg/dL), as shown in the PBS (control) group, were elevated up to 315.7 ± 7.5 mg/dL, 170.2 ± 2.2 mg/dL, and 174.1 ± 4.9 mg/dL, respectively, by the CML treatment (Figure 7A–C). Exposure to OOO proved effective in reducing CML-elevated TG levels; however, it remains ineffective in significantly reducing elevated TC and LDL-C levels. In contrast, treatment of OSO effectively reduced the elevated TC, TG, and LDL-C levels by 1.2-fold (266.6 ± 1.2 mg/dL), 1.4-fold (120.7 ± 1.8 mg/dL), and 1.7-fold (103.7 ± 6.6 mg/dL), respectively, compared with the CML-treated group. A substantially diminished HDL-C (107.6 ± 7.4 mg/dL) level was detected in the CML-treated group compared to the PBS (control, 141.4 ± 0.2 mg/dL) group (Figure 7D). The CML-diminished HDL-C level was significantly increased to 138.8 ± 5.7 mg/dL by the OSO treatment, whereas a non-significant increase in the CML-compromised HDL-C level was observed in the OOO-treated group. A substantially lower HDL-C/TC ratio was observed in response to CML, which was significantly enhanced by OSO treatment (Figure 7E). However, no significant effect of OOO was observed on the elevation of the CML-compromised HDL-C/TC ratio.

A significantly elevated blood glucose level was detected in the CML-injected group, which was significantly reduced to ~2-fold by treatment with OOO and OSO, respectively (Figure 7F).

### 3.9. Antioxidant Ability in Plasma

**Figure 8:**
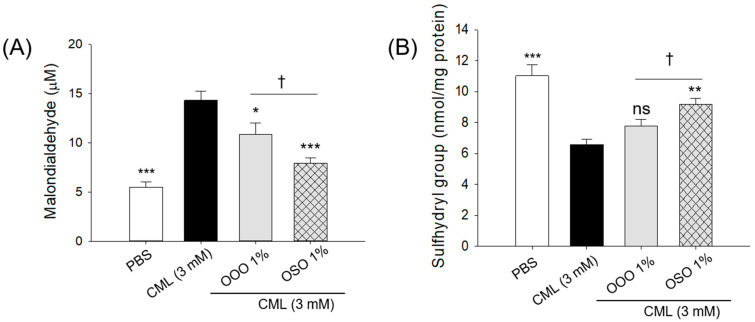

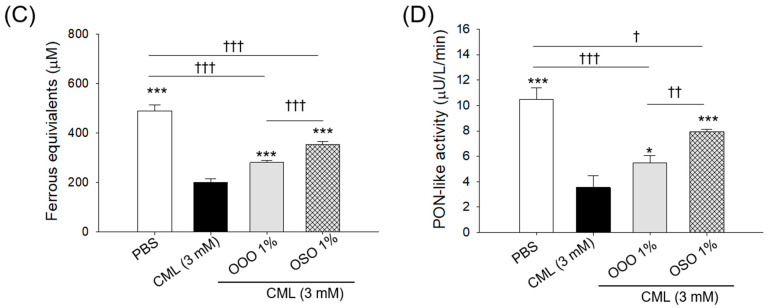
Comparison of plasma (**A**) malondialdehyde (MDA), (**B**) sulfhydryl content, (**C**) ferric ion reduction activity (FRA) and (**D**) paraoxonase (PON) activity of olive oil (OO)-, sunflower oil (SO)-, ozonated olive oil (OOO)- and ozonated sunflower oil (OSO)-treated zebrafish. Each point in the bar graph represents the mean ± SEM from four (*n* = 4) independent experiments. The statistical differences *p* < 0.05 (*), *p* < 0.01 (**) and *p* < 0.001 (***) correspond to the CML-injected group using one-way ANOVA following Tukey’s post hoc analysis. *p* < 0.05 (^†^), *p* < 0.01 (^††^), and *p* < 0.001 (^†††^) represent the statistical difference between the marked groups determined by the *t*-test; a non-significant difference is depicted by “ns”.

Compared with the PBS (control) group, a significantly higher MDA level (2.1-fold) was detected in the CML-injected group (Figure 8A). Treatment with both OOO and OSO substantially reduced the CML-elevated MDA level. In contrast to the elevated MDA level, the plasma sulfhydryl content was significantly reduced by 1.5-fold in the CML-treated group compared to the PBS (control) group (Figure 8B). A significantly ~1.2-fold enhanced sulfhydryl content was observed in the OOO- and OSO-treated groups compared to the CML-treated group.

Compared with the PBS (control) group, plasma FRA and PON-like activities were significantly reduced following exposure to CML (Figure 8C,D). The CML-diminished FRA and PON activities were significantly enhanced by treatment with OOO and OSO by 1.4-, 1.8-, 1.6-, and 2.2-fold. However, compared with OOO, ~1.4-fold higher FRA and PON activities were observed in the OSO-treated group, attesting to the functional superiority of OSO over OOO.

### 3.10. Hepatic Histology

**Figure 9:**
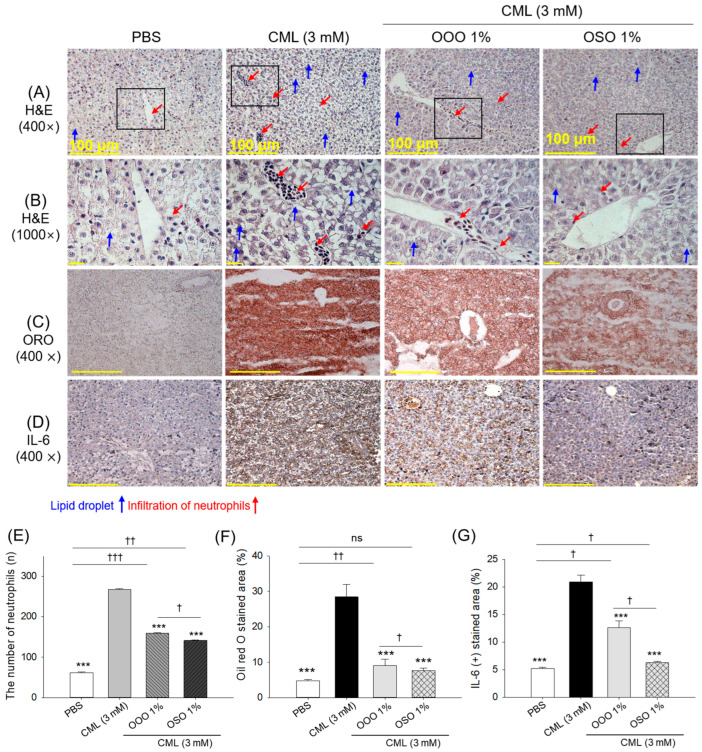
Comparative effects of olive oil (OO), sunflower oil (SO), ozonated olive oil (OOO), and ozonated sunflower oil (OSO) on liver histology of carboxymethyllysine (CML)-treated zebrafish. (**A**) Hematoxylin and eosin (H&E) staining (400× magnification). (**B**) A 1000× magnified view of the H&E section covered by the black box in image A. Blue and red arrows indicate the oil droplets and neutrophils, respectively. (**C**) Oil Red O (ORO)-stained section. (**D**) Immunohistochemical (IHC) staining for the detection of interleukin (IL)-6. (**E**, **F, G**) Quantification of neutrophils, ORO, and IL-6-stained area, respectively. For quantitative analysis, four sections and five random fields/sections from each group were examined under a microscope. The statistical difference *p* < 0.001 (***) corresponds to the CML-injected group using one-way ANOVA following Tukey’s post hoc analysis. *p* < 0.05 (^†^), *p* < 0.01 (^††^), and *p* < 0.001 (^†††^) represent the statistical difference between the marked groups determined by the *t*-test; a non-significant difference is depicted by “ns”.

Hepatic H&E staining (Figure 9 A,B,E) revealed intense neutrophil infiltration and lipid droplet accumulation in the CML-treated group that was notably 4.3-fold higher than the neutrophil counts observed in the PBS (control) group. Treatment with OOO and OSO effectively countered CML-induced hepatic inflammation, as reflected by neutrophil counts that were 1.6- and 1.9-fold lower in the OOO- and OSO-treated groups than in the CML-treated groups.

ORO staining revealed high lipid accumulation in the CML-treated group, which was, significantly, 5.9-fold higher than that of the ORO-stained area observed in the PBS (control) group (Figure 9C,F). The CML-elevated ORO-stained area was, substantially, 3.1-fold and 3.9-fold reduced by the treatment with OOO and OSO. Likewise, IL-6 production was 3.9-fold higher in the hepatic section of the CML-treated group than in the PBS group (Figure 9D,G). Notably, reductions of 1.6-fold and 3.3-fold in IL-6 production compared to the CML-treated group were observed in the OOO- and OSO-treated groups, suggesting the impact of both ozonated oils on hepatic inflammation. Both OOO and OSO were found to be effective in preventing CML-induced hepatic damage; however, compared to OOO, OSO treatment showed significantly higher (*p* < 0.05) reductions in neutrophil infiltration, ORO-stained area, and IL-6 production, by 1.2-fold, 1.2-fold, and 2-fold, respectively. This suggests that OSO has greater functionality than OOO in mitigating CML-induced changes in the liver.

### 3.11. Reactive Oxygen Species and Cellular Senescence in the Liver

**Figure 10:**
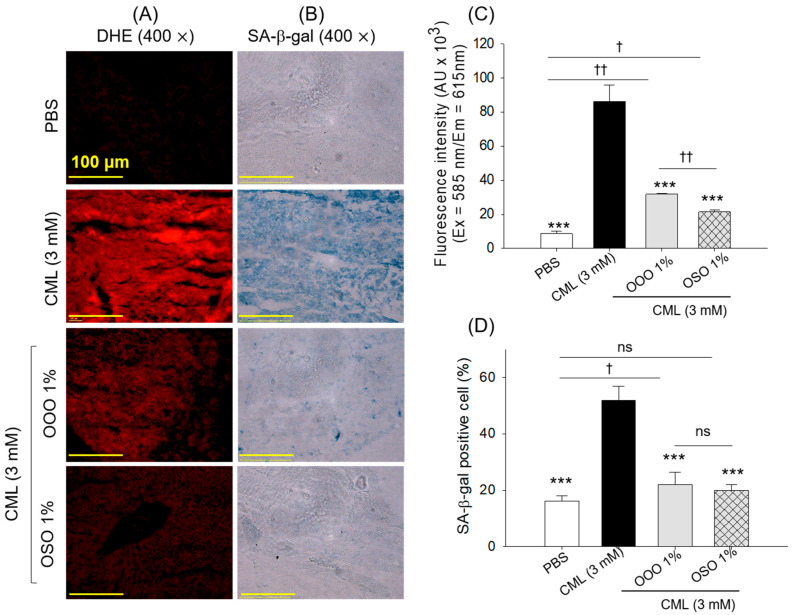
The comparative effects of olive oil (OO), sunflower oil (SO), ozonated olive oil (OOO), and ozonated sunflower oil (OSO) on the reactive oxygen species (ROS) and senescence in the livers of carboxymethyllysine (CML)-treated zebrafish. (**A**) Dihydroethidium (DHE) fluorescent staining. (**B**) Senescent-associated β-galactosidase (SA-β-gal) staining. (**C**,**D**) Quantification of DHE fluorescent intensity and senescent positive cells. For quantitative analysis, four sections and five random fields from each group were examined under a microscope. The statistical difference *p* < 0.001 (***) corresponds to the CML-injected group using one-way ANOVA following Tukey’s post hoc analysis. *p* < 0.05 (^†^) and *p* < 0.01 (^††^) represent the statistical difference between the marked groups determined by the *t*-test; a non-significant difference is depicted by “ns”.

DHE fluorescent staining (Figure 10A,C) and senescent-associated β-galactosidase (SA-β-gal) staining (Figure 10B,D) revealed 9.9-fold and 3.2-fold higher ROS and cellular senescence in the CML-injected group than in the PBS (control) group. In contrast, OOO and OSO significantly reduced CML-elevated ROS and senescence. Compared with OOO, the OSO treatment showed a 1.5-fold reduction in ROS levels; however, no significant difference between OOO and OSO was observed in mitigating CML-triggered cellular senescence.

### 3.12. Hepatic Damage Biomarkers in Blood

**Figure 11:**
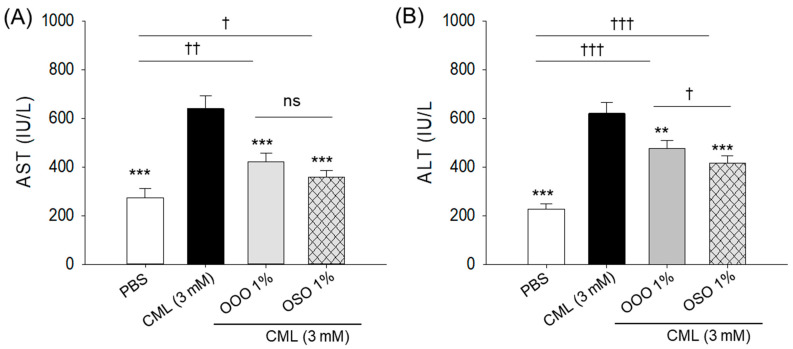
Comparative plasma (**A**) aspartate aminotransferase (AST) and (**B**) alanine aminotransferase (ALT) levels of zebrafish treated with olive oil (OO), sunflower oil (SO), ozonated olive oil (OOO), and ozonated sunflower oil (OSO) under the influence of carboxymethyllysine (CML). Each point in the bar graph represents the mean ± SEM value obtained from four (*n* = 4) independent experiments. The statistical differences *p* < 0.01 (**) and *p* < 0.001 (***) correspond to the CML-injected group using one-way ANOVA following Tukey’s post hoc analysis. *p* < 0.05 (^†^), *p* < 0.01 (^††^), and *p* < 0.001 (^†††^) represent the statistical difference between the marked groups determined by the *t*-test; a non-significant difference is depicted by “ns”.

A 2.3-fold and 2.7-fold increase in blood AST and ALT levels, respectively, compared to the PBS control group, was observed in the CML-injected group (Figure 11). The CML-elevated AST and ALT levels were significantly reduced by 1.5~1.8-fold and 1.2~1.5-fold following treatment with OOO and OSO, respectively. However, compared with OOO, the OSO treatment showed notably lower AST and ALT levels (~1.2-fold lower), suggesting that OSO is more potent than OOO at protecting the liver.

### 3.13. Histological Analysis of Kidneys

**Figure 12:**
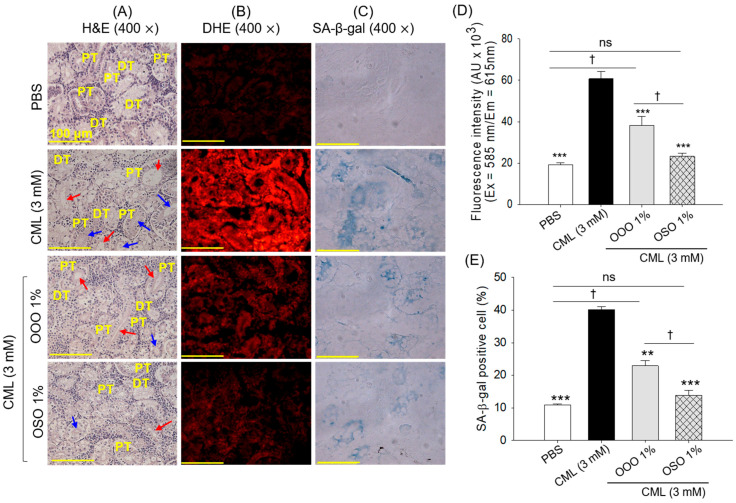
Comparative effect of olive oil (OO), sunflower oil (SO), ozonated olive oil (OOO), and ozonated sunflower oil (OSO) on kidney histology of carboxymethyllysine (CML)-treated zebrafish. (**A**) Hematoxylin and eosin (H&E) staining; PT and DT are abbreviations for proximal tubule and distal tubule, respectively; red and blue arrows depict dilated tubular lumen and cellular debris in tubular cast, respectively. (**B**) Dihydroethidium (DHE) fluorescent staining and (**C**) senescent-associated β-galactosidase (SA-β-gal) staining. (**D**) and (**E**) Quantification of DHE fluorescent intensity and SA-β-gal cells, respectively. For quantitative analysis, four sections and five random fields from each group were examined under a microscope. The statistical differences *p* < 0.01 (**) and *p* < 0.001 (***) correspond to the CML-injected group using one-way ANOVA following Tukey’s post hoc analysis. *p* < 0.05 (^†^) represents the statistical difference between the marked groups determined by the *t*-test; a non-significant difference is depicted by “ns”.

The kidney H&E staining revealed disrupted distal (DT) and proximal tubular (PT) structures, with an enlarged tubular lumen and cellular debris in the CML-injected group (Figure 12A). In contrast, in the PBS-injected (control) group, well-defined and properly arranged PT and DT structures devoid of elevated tubular lumen and debris were observed. Compared with the CML group, treatment with ozonated oils, specifically OSO, exhibited a substantial kidney-protective effect, as evidenced by well-organized DT and PT structures. Also, OOO protects against CML-induced kidney damage by restoring PT and DT morphology; however, dilated lumens and cellular debris in tubular casts are observed in some regions.

DHE fluorescence staining showed the highest ROS level in the CML-injected group, which was substantially reduced to 1.6- and 2.6-fold following treatment with OOO and OSO, respectively (Figure 12B,D). However, OSO treatment showed a significantly lower 1.6-fold reduction in ROS compared to the OOO-treated group. Consistent with the DHE fluorescent staining, SA-β-gal staining revealed 1.8-fold and 2.9-fold lower cellular senescence in the OOO and OSO treated groups, respectively, compared to the CML-injected group (Figure 12C,E). Compared with OOO, a notable 1.6-fold reduction in senescent-positive cells in the OSO group suggests greater effectiveness of OSO than OOO in preventing CML-induced senescence.

## 4. Discussion

SO and OO are edible vegetable oils frequently used for ozonation. In 1896, Tesla invented an electric apparatus for ozone generators to bubble ozone gas in olive oil via the Tesla Ozone Company [32]. SO and OO differ markedly in their fatty acid compositions, leading to the formation of distinct ozonation products. SO is rich in polyunsaturated fatty acids, with linoleic acid comprising ~69% of its total fatty acids, followed by monounsaturated oleic acid (20%) [33]. It also contains high levels of vitamin E, predominantly as α-tocopherol (~90%) [34]. In contrast, OO is primarily composed of monounsaturated oleic acid (65–85%) and contains α-tocopherol and phenolic compounds [35]. These compositional differences of SO and OO influence the physicochemical characteristics of their corresponding ozonated oils (OSO and OOO). In a comparative evaluation between ozonated oils, OSO exhibited higher peroxide (382 meqO_2_/kg) and acid value (8.9 mg KOH/g) than OOO (240.5 meqO_2_/kg and 3.6 mg KOH/g, respectively) [36], suggesting that the compositional difference between these two ozonated oils resulted in different peroxide and acid values and consequently different functionality. Despite the individual functionality of both OOO and OSO being reported, comparative studies between these two ozonated oils remain limited. In the present work, two ozonated oils (OSO and OOO) were compared in vitro and against CML-derived adverse events in zebrafish. Compared with SO and OO, the ozonated oils (OOO and OSO) demonstrated distinct optical properties, including a noticeable shift in l_max_, indicating ozone-induced modification at the unsaturated site and the formation of noble oxygen-containing species. These observations were consistent with the e-nose outcomes, which revealed the generation of unique volatile constituents along with the enhanced concentrations of many compounds already present in the non-ozonated oils, confirming extensive chemical transformation during the ozonation process. The in vitro antioxidant and embryo-protective activities revealed that, compared to OO and SO, their ozonated samples exhibited higher radical-scavenging activity and a significant enhancement in HDL-associated PON and FRA activities, underscoring a positive impact of ozonation on the functional enhancement of these oils.

Furthermore, OSO demonstrated significantly higher in vitro antioxidant activity than OOO, highlighting the critical influence of source material on the biological efficacy of ozonation. So far, few studies have documented the comparative bio-functionality of ozonated oils. However, a few studies have compared the effects of OOO and OSO and have reported higher antimicrobial activity for OSO than OOO against a variety of bacterial and fungal species [36]. Notably, marked fluorescence quenching related to the tryptophan residue was observed in HDL treated with OSO, followed by OOO, while the SO-treated group exhibited the least quenching effect. The elevated hexanal level detected in OSO (as observed by e-nose analysis) substantially contributes to the enhanced quenching. The observation is in good agreement with previous reports demonstrating that hexanal can interact with amino acid residues, including tryptophan, resulting in fluorescence quenching via non-covalent and covalent bonding [37].

Consistent with the in vitro antioxidant findings, the in vivo study in zebrafish embryos suggests that ozonated samples (OSO and OOO) are more potent than the non-ozonated oils in mitigating CML-triggered toxicity. Moreover, the higher effect of OSO over OOO is attributed to its higher antioxidant activity, which has been recognized as a key driver of a variety of protective events that prevent apoptosis [38,39] and developmental defects in zebrafish embryos [40,41] induced by external oxidative stress. Similar to the beneficial effects observed in zebrafish embryos, treatment with both OOO and OSO markedly protected adult zebrafish against CML-induced swimming impairment and acute mortality. The antioxidant and anti-inflammatory effects of OOO [17,42,43] and OSO [14,16] are the primary reasons for their preventive effects. This notion is consistent with earlier reports that depict inflammation as a key culprit behind swimming abnormalities and acute death in zebrafish [44]. Also, studies show that inhibiting proinflammatory mediators such as IL-6 and TNF-α in zebrafish results in higher survivability [44], highlighting a strong association between inflammation and acute death. Nevertheless, further studies are needed to elucidate the detailed molecular mechanism by which ozonated oil prevents acute mortality and swimming impairment in zebrafish.

CML has been recognized as inducing oxidative stress and impairing antioxidant defense [45]. Likewise, high MDA levels and reduced FRA, which are key markers of lipid oxidation [46] and total antioxidant activity [47], respectively, were observed in the CML-treated groups. Also, a diminished sulfhydryl, a primary antioxidant against peroxyl radicals [48,49], was noticed in the plasma of the CML-treated group. The elevated MDA levels and diminished FRA and sulfhydryl contents were substantially reduced in the OOO- and OSO-treated groups, attesting to their strong antioxidant potential against CML-induced oxidative stress. In addition, diminished PON-like activity was observed in the CML-treated group, which was substantially augmented following treatment with OOO and OSO. However, relative to OOO, the OSO treatment proved more effective in augmenting the antioxidant defense system, underscoring the functional benefit of OSO over OOO. So far, to the best of our knowledge, there is no comparative antioxidant study comparing OOO and OSO; however, individual studies have assessed their functionality in various animal models [50]. The cellular antioxidant potential of OOO has been attributed to its ability to modulate key antioxidant enzymes, including SOD, Cat, and glutathione peroxidase (GSH-px), in mice [50], as well as to improvements in PON activity in rats [51]. Similarly, OSO cellular antioxidant potential has been recognized in a variety of animal models [52,53]. In fact, this study commenced with purified HDL, suggesting a time-dependent positive effect of OSO on augmenting HDL-associated PON activity [16], which was further shown to improve PON activity when tested in mice [53], strengthening the present outcomes.

The pro-inflammatory effect of CML is among the key factors that lead to dyslipidemia, as several studies have documented positive associations between inflammation and oxidative stress and metabolic disorders [54] and dyslipidemia [55]. Similarly, a disturbed plasma lipid profile was observed in the CML-treated group and was substantially mitigated by OSO treatment. The role of OSO has been substantially linked to dyslipidemia [56]; however, the effect of OOO has not been extensively studied. Nevertheless, a meta-analysis described a non-significant effect of OOO on the plasma lipid profile in diabetic patients [17], supporting the present findings. The findings showed a clear disparity between the actions of OSO and OOO in preventing CML-triggered dyslipidemia.

CML has been recognized to cause hepatic inflammation, and the induction of inflammatory markers consequently causes hepatic damage [57]. Likewise, high inflammation, oxidative stress, and fatty liver were observed in the CML-injected group, which were mitigated by exposure to OOO and OSO. The anti-inflammatory properties of OOO [17,42] and OSO [53,56] likely play a crucial role in mitigating CML-induced liver damage, as inflammation is a major contributor to the development and progression of hepatic injury [58]. In addition, the reduction in ROS levels observed following OOO and OSO treatment can further protect the liver, given that excessive ROS generation is well known to promote liver toxicity, oxidative stress, and fatty liver [59,60]. A direct free radical scavenging ability, along with the cellular antioxidant ability of OOO [43,50] and OSO [14,16,53], is the key event for preventing CML-imposed ROS generation and liver damage. The improved liver health in the OOO- and OSO-supplemented groups is also evidenced by reduced AST and ALT levels, which are recognized as important hepatic function biomarkers [61].

Similar to the effect observed in the liver, both OOO and OSO substantially protect against kidney damage and mitigate ROS generation induced by the CML. Also, reduced kidney senescence in response to OOO and OSO was observed. Reduced senescence in response to OOO and OSO supplementation can be attributed to lower ROS levels in these groups. This statement is in line with the literature showing a direct relationship between ROS and the onset of senescence [62,63]. Compared to OOO, OSO has substantially greater effects in reducing ROS and senescence, owing to its higher antioxidant potential. The elevation of CML compromised plasma sulfhydryl levels in OOO and OSO, also indicating improved kidney health in these groups, as diminished sulfhydryl levels are correlated with inflammatory conditions and kidney damage [64,65].

Limitations of the study: This in vitro study evaluating the effect of ozonated and non-ozonated oils on WMF and antioxidant activities was conducted using human-derived HDL rather than zebrafish-derived HDL. This approach was chosen because zebrafish have a small blood volume (~7 μL), and the total blood volume obtained from zebrafish (*n* = 40/group) was insufficient for HDL isolation. Future work will employ a large number of zebrafish to obtain sufficient blood volume for HDL isolation. Furthermore, future investigations will focus on elucidating the molecular mechanisms underlying the effects of OSO and OOO, particularly their regulation of the NF-κB and Nrf2 signaling pathways, to establish a clear mechanistic basis for their biological activities.

## 5. Conclusions

Ozonated oils (OSO and OOO) exhibited markedly different functionality compared with their non-ozonated counterparts (SO and OO). Among the ozonated oils, OSO demonstrated significantly higher antioxidant capacity and exerted a stronger positive impact on HDL-associated PON and FRA activity than OOO. In adult zebrafish, both OSO and OOO alleviated CML-induced oxidative stress and increased the HDL-C/TC ratio, thereby improving swimming recovery and survivability. Furthermore, both OSO and OOO protect the liver and kidneys; however, OSO, compared with OOO, provides notably better protection against CML-induced hepatic and renal damage by reducing inflammation, ROS production, and cellular senescence. Collectively, the findings indicate that both OSO and OOO have a protective effect in mitigating CML-induced pathological events by modulating oxidative stress, inflammation, and HDL function, thereby protecting the liver and kidneys. However, compared to OOO, OSO exhibits a slightly greater effect. The current findings are based on preclinical studies conducted in zebrafish; therefore, further validation in mammalian models and clinical studies is required to establish the relevance of OSO and OOO safety and efficacy in humans.

## Figures and Tables

**Table 1:** Density, pH, and wavelength maximum of olive oil, sunflower oil, and their ozonated products.

Sample Type	Density (g/mL)	pH	Wavelength Maxima	Wavelength Shift ^1^
OO	0.911	4~5	208 nm	~28 nm (red shift)
OOO	0.913	<3	236 nm	
SO	0.913	4~5	232 nm	~16 nm (blue shift)
OSO	0.915	3~4	215 nm	

Abbreviations: OO: olive oil, OOO: ozonated olive oil, SO: sunflower oil, OSO: ozonated sunflower oil. ^1^ Wavelength shift indicates the difference between the wavelengths of the oils before and after ozonation.

**Table 2:** Comparison of the volatile compounds in the SO, OO, OSO, and OOO using an electronic nose.

RT (Sec)		Peak Area				Compounds	Sensory Description
MXT-5	MXT-1701	SO	OSO	OO	OOO		
21.3	–	20,5990 ± 475	–	1791 ± 150	393,314 ± 6428	Methanethiol	Cheese, cooked cabbage, fish, garlic, meaty, rotten egg
45.76	–	266 ± 12	4926 ± 111	118 ± 11	31,704 ± 539	Heptane	Alkane, fruity, sweet
54.10	–	–	–	–	521 ± 88	2-Ethyl furan	Acidic, chemical, pungent, rubber, sweet
60.18	–	–	–	–	6256 ± 300	1,3-Dichloro-propane	
63.17	–	14 ± 28	225 ± 16	–	712 ± 11	Hexan-2-one	Cinnamon, ethereal, fruity
71.21	–	657 ± 26	509,525 ± 11,320	317 ± 35	386,941 ± 3821	Hexanal	Acorn, fatty, fishy, fruity, grassy, leafy
83.63	–	–	166 ± 146	–	859 ± 871	2,4-Dimethyl-1,3-dioxane	
–	18.96	20,100 ± 457	367,131 ± 9431	1057 ± 124	190,809 ± 3887	Methanethiol	Alkane, ethereal, kerosene
–	23.72	206 ± 10	3512 ± 101	519 ± 13	139,002 ± 23,13	3-Methylpentane	Alkane, fruity, sweet
–	24.69	286 ± 22	31,897 ± 808	405 ± 22	236,190 ± 3380	Hexane	Almond, herbaceous, malty, pungent, rubber
–	37.68	258 ± 25	883 ± 147	–	8194 ± 121	Heptane	Fruity, plastic, pungent, rubber
–	45.43	228 ± 26	426 ± 37	250 ± 215	4328 ± 733	2-Ethyl furan	Fruity, plastic, pungent, rubber, sweet
–	56.26	176 ± 14	11,868 ± 241	23 ± 45	34,611 ± 434	Pentanal	Almond, herbaceous, malty, pungent, rubber
–	59.32	105 ± 26	53,563 ± 1256	119 ± 64	347,133 ± 4140	Penta-2-ol	Fruity, plastic, pungent, sweet
–	69.38	–	157 ± 161	–	15,432 ± 185	3-Pentanol	Fruity, green
–	73.54	–	5218 ± 102	–	11,397 ± 168	(E)-3-Hexanal	Green
–	84.65	753 ± 12	564,097 ± 10,996	249 ± 33	308,625 ± 3125	Hexanal	Acorn, fatty, fishy, fruity, grassy, herbaceous, leafy

The values represent the mean ± SEM obtained from five independent experiments (*n* = 5) for each group. Abbreviations: SO: sunflower oil, OSO: ozonated sunflower oil, OO: olive oil, OSO: ozonated olive oil, RT: retention time. MXT-5 and MXT-1701 represent two different capillary columns.

## Data Availability

The original contributions presented in this study are included in the article/Supplementary Material. Further inquiries can be directed to the corresponding author.

## Supplementary Materials

The following supporting information can be downloaded at https://www.mdpi.com/article/10.3390/antiox15070840/s1, Table S1: Certificate of ozonated sunflower oil (OSO) quality analysis; Table S2: Certificate of water quality analysis; Supplementary Material Section S1: Method to quantify plasma lipoproteins and hepatic function biomarkers; Supplementary Material Section S2: Malondialdehyde (MDA), sulfhydryl group, ferric ion reduction (FRA) activity and paraoxonase (PON) activity; Figure S1: UV spectrum (scan range 190–350 nm) of the sunflower oil (SO), ozonated sunflower oil (OSO), olive oil (OO) and ozonated olive oil (OO); Figure S2: A comparative effect of olive oil (OO), sunflower oil (SO), ozonated olive oil (OOO), and ozonated sunflower oil (OSO) on the carboxymethyllysine (CML) induced toxicity in zebrafish embryos. (A) Survivability kinetics (0–72 hr). Each data point represents the mean ± SEM obtained from 150 embryos divided into three groups (n = 3, 50 × 3 = 150). (B) Embryos survivability at 72 hr post treatment. (C) Images of embryos post 24, 48, and 72 hr treatment. The red arrow indicates dead embryos. (D) Representative images depicting somites. (E) and (F) dihydroethidium (DHE) and acridine orange (AO) staining, respectively. (G) and (H) Quantification of average somite counts and fluorescent intensity, respectively. The statistical difference (*p* < 0.001; ***) was determined using one-way ANOVA with Tukey’s post hoc analysis for the CML-injected group. The *p* < 0.05 (^†^) and *p* < 0.01 (^††^) represent the statistical difference between the marked groups determined by *t*-test. A non-significant difference is depicted by “ns”; Figure S3: The comparative effect of olive oil (OO), sunflower oil (SO), ozonated olive oil (OOO), and ozonated sunflower oil (OSO) on the CML-triggered adverse events in zebrafish embryos. (A) Heartbeat calculated at 72 hr post treatment. Each point represents the mean ± SEM from three (*n* = 5) independent experiments. (B) Pictorial view of the representative embryos at 144 hr post-treatment. Blue arrows highlight the uninflated swimming bladder, green and red arrows represent yolk sac edema and pericardial edema, respectively, while brown and black arrows represent tail fin curvature and back bending, respectively. The statistical difference *p* < 0.001 (***) corresponds to the CML-injected group using one-way ANOVA following Tukey’s post hoc analysis. The *p* < 0.05 (^†^) and *p* < 0.01 (^††^) represent the statistical difference between the marked groups determined by the *t*-test.
