# Supplementary material for: Comparison of HDL-Associated Antioxidant Activities and Anti-Inflammatory Effect Between Ozonated Sunflower Oil (OSO) and Ozonated Olive Oil (OOO) Under Carboxymethyllysine-Induced Acute Phase in Zebrafish Adults and Embryos
Source: Antioxidants (Basel). 2026 Jul 3;15(7):840. doi: 10.3390/antiox15070840 (PMC13404453; doi:10.3390/antiox15070840)
Supplement: Supplementary file 1 [file antioxidants-15-00840-s001.zip › antioxidants-4395193-supplementary.pdf]

Supplementary material

Supplementary Table S1

CNIC
CENTRO NACIONAL DE INVESTIGACIONES CIENTÍFICAS

Ave 25 No. 15202, entre 21 y 21ª, Cubanacán, Playa, La Habana, República de Cuba.
Sitio: www.cnic.cu / E-mail: cnic.cuba@cnic.cu
Teléfono: 7 215 21 83

NC-ISO 9001:2015
NC-ISO 14001:2015
NC 136:2017
Registro No. 004-2024

Results of QUALITY CONTROL (ORAL OLEOZON)

**Characteristics**		**Acceptance Limits**	Batch no. **(2309066-O)**
Organoleptics	Appearance	Emulsion<br>Free from foreign matter	Fulfills
	Color	Light milky yellow	Fulfills
	Odour	Caractheristic	Fulfills
Peroxide Index		500 – 800mmol-equiv/kg	746.14
Acidity		<25 mg KOH/g	2.45
Aldehides Concentration		< 0.9 mmol/g	0.24
Viscosity		90 - 600 mPa.s	110.35
Microbial Limits	Aerobic microorganisms	< 10^3CFU/plaque	≤ 10
	Fungi	< 10^2CFU/plaque	No presence
	Escherichia coli<br>*Entero bacterias, S. aureus, P. eruginosa, Candida. Sp.*	Absence in 1g	Absence
	Salmonella	Absence in 10g	

Supplementary Table S1: Certificate of ozonated sunflower oil (OSO) quality analysis

Supplementary Table S2

Original certificate of water quality

분석과학을 선도하는 기업

KIRIM (주)기림생명과학원

KiRim Institute of Life Science Co.,Ltd.

받 음: 대구 동구 동내로 76 (동내동)
한국메디벤처센터 지하2층 관리실 귀하

41061

수질검사성적서

수신 : 한국메디벤처센터
제목 : 먹는물(저수조수) 수질검사성적서 발급

발 급 일 : 2025년 5월 2일
발급번호 : KWCT3379961
발 신 : (주)기림생명과학원

「먹는물 수질기준 및 검사 등에 관한 규칙」 제3조제2항에 따라 다음과 같이 먹는물 수질검사성적서를 발급합니다. 이 성적서는 의뢰인이 제시한 시료에 대한 결과이며 검사목적 이외에는 사용할 수 없습니다.

1. 시료 내용

접수번호	12506438	접 수 일	2025년 4월 28일
의 뢰 인	한국메디벤처센터		
시 료 명	저수조수		
검사목적	수도법(저수조수 수질검사)		
채수장소	대구 동구 동내로 76 (동내동)		
시료채취	기술인력채취 (주)기림생명과학원 성기현 채수일시 : 2025.04.28		
비 고	판정은 수도법 시행규칙 제22조4의⑤항 및 ⑦항에 따른 먹는물 수질기준 및 검사등에 관한 규칙[별표1]에 의거합니다.		

2. 수질검사 결과

검사항목	기준	검사결과
탁도	0.5 NTU 이하	0.16
수소이온농도	5.8 ~ 8.5	7.3
잔류염소	0.1 ㎎/L이상 4.0 ㎎/L이하	0.18
일반세균	100 CFU/mL이하	0
총대장균군	불검출/100mL	불검출
분원성대장균군	불검출/100mL	불검출
종합결과	적합	

• 탁도(등급외 성능),잔류염소(1등급)는 간이측정기를 사용하여 측정함.

(주)기림생명과학원 대표이사

- 환경과 생명을 소중히 하는 아름다운 기업 -

Certificate of water quality (translated in English)

Drinking Water Quality Test Report
Recipient: Korea Mediventure Center
B2 Management Office
76 Dongnae-ro, Dong-gu, Daegu (Dongnae-dong), 41061
Issued by: Kirim Life Science Co., Ltd.
Issue Date: May 2, 2025
Report Number: KWCT3379961
Subject: Drinking Water (Reservoir Water) Quality Test Report

Basis of Issuance
This report is issued in accordance with Article 3, Paragraph 2 of the *Regulations on Drinking Water Quality Standards and Testing*.
It presents the results of the sample provided by the client. The report must not be used for purposes other than water quality testing.

1. Sample Information
- **Receipt Number:** 12506438
- **Receipt Date:** April 28, 2025
- **Client:** Korea Mediventure Center
- **Sample Name:** Reservoir Water
- **Purpose of Test:** Waterworks Act (Reservoir Water Quality Test)
- **Sampling Location:** 76 Dongnae-ro, Dong-gu, Daegu (Dongnae-dong)
- **Collected by:** Technical staff (Seong Ki-hyun, Kirim Life Science Co., Ltd.)
- **Sampling Date/Time:** April 28, 2025
- **Remarks:** Evaluation conducted in accordance with Article 22, Paragraph 4 of the Enforcement Rules of the Waterworks Act and Annex 1 of the *Regulations on Drinking Water Quality Standards and Testing*.

2. Test Results

Test Item	Standard	Result
Turbidity	≤ 5 NTU	0.16
pH (Hydrogen Ion)	5.8 – 8.5	7.3
Residual Chlorine	0.1 – 4.0 mg/L	0.18
General Bacteria	≤ 100 CFU/mL	0
Total Coliform Group	Not detected / 100 mL	Not detected
Fecal Coliform Group	Not detected / 100 mL	Not detected

Comprehensive Result: Suitable (Compliant)

Note: Turbidity (non-graded performance) and residual chlorine (Grade 1) were measured using a simplified measuring device.

Certification
Issued by: **Kirim Life Science Co., Ltd.** Representative Director
- A beautiful company that values environment and life -

Supplementary Table S2: Certificate of water quality analysis.

Section S1.

1. Method to quantify plasma lipoproteins and hepatic function biomarkers

The plasma total cholesterol (TC) and triglycerides (TGs) were determined using commercial assay kits (cholesterol, AM 202-K, and TGs, AM 157-K, Asan Pharmaceutical, Hwasung, Republic of Korea) as per the method suggested by the suppliers. In brief, 5 μL serum was mixed with 200 μL reaction mixture (supplied with a commercial assay kit) for the TC analysis. The content was incubated at 37°C for 10 min, resulting in a red-colored product quantified by adsorption at 490 nm (Microplate reader, iMark™ Bio-Rad, Hercules, CA, USA).

Similarly, 5 μL serum was mixed with a 200 μL of TGs-specific reaction mixture (supplied with a commercial assay kit) for TGs analysis. The content was incubated for 10 min at 37°C, and the formed colored product was quantified by taking adsorption at 490 nm.
For HDL-C analysis, serum was mixed in an equal ratio with the separation solution (supplied with a commercial assay kit), followed by centrifugation at 3,000 rpm for 10 min at 25°C. The supernatant (20 μL) was collected and blended with a 200 μL reaction mixture (supplied with a commercial assay kit). After 10 min incubation at 37°C, red color intensity corresponding to HDL-C was quantified by taking absorption at 490 nm (Microplate reader, iMark™ Bio-Rad, Hercules, CA, USA).
The LDL-C level was quantified using the Friedewald equation:

$$LDL\text{-}C = TC - HDL\text{-}C - (TG/5)$$

The commercial diagnostic kit (AM102K and AM103-K, Asan Pharmaceutical, Hwasung, Republic of Korea) was used to quantify aspartate transaminase (AST) and alanine transaminase (ALT) levels in the plasma, following the instructions suggested by the manufacturers. Briefly, 5 μL of plasma was combined with 250 μL of either AST or ALT-specific solution, as supplied in the diagnostic kit. Following a 30 min incubation for AST or 60 min incubation of ALT at 37°C, the mixture was then blended with 250 μL of the respective coloring reagent (AST or ATL-specific, provided in the diagnostic kit). After a subsequent 20 min incubation at room temperature, 250 μL of 0.4 N NaOH was introduced to halt the reaction. Finally, the AST and ATL were quantified by measuring absorbance at 490 nm (Microplate reader, iMark™, Bio-Rad, Hercules, CA, USA).

Section S2.

2. Malondialdehyde (MDA), sulfhydryl group, ferric ion reduction (FRA) activity and paraoxonase (PON) activity

The blood malondialdehyde (MDA) level was quantified by mixing plasma sample (20 μL, equivalent to 1 mg/mL protein) with trichloroacetic acid (50 μL, 0.2 mg/μL, pH 1.4) and thiobarbituric acid (100 μL, 6.7 μg/μL, pH 2.3). Following a 10-min incubation at 95 °C, the absorbance at 560 nm was recorded.

The sulfhydryl group was quantified by mixing 60 μL of plasma (1 mg/mL protein) with 60 μL of 5,5′-dithio-bis-(2-nitrobenzoic acid) (DTNB) (4 mg/mL). After 12 hr incubation at room temperature, absorbance 412 nm was determined, and sulfhydryl groups were quantified utilizing 13,600 M^{-1} cm^{-1} extinction coefficient (ε) of DTNB.

To assess ferric ion reduction (FRA) capacity, 20 μL of the plasma (1 mg/mL equivalent protein) was mixed with 180 μL of FRA reagent (prepared by blending 10 mL of acetate buffer (0.2M, pH 3.6) with 1.25 mL each of 2,4,6-tripridyl-S triazin (10 mM, pH 1.9) and $FeCl_3$ (20 mM, pH 2.1). After incubating the mixture at RT for 60 min, absorbance was measured at 593 nm. The results were quantified in μM ferric equivalents based on a ferrous sulfate standard curve.

For paraoxonase activity 20 μL of plasma (1 mg/mL equivalent protein) was mixed with 180 μL of buffer (pH 8.3) [Tris-HCl (90 mM), NaCl (3.6 mM), $CaCl_2$ (90 mM)] containing the paraoxon-ethyl substrate (0.55 M). After 60 min incubation at 25°C, an absorbance (415 nm) was recorded using Microplate reader (Model, iMark™ S.N 21275, Bio-Rad, Hercules, CA, USA) to quantify the production of *p*-nitrophenol, a hydrolysis product of paraoxon-ethyl. Results are expressed as μU/L/min employing the extinction coefficient 17,000 $M^{-1}cm^{-1}$ for *p*-nitrophenol.

Supplementary Figure S1

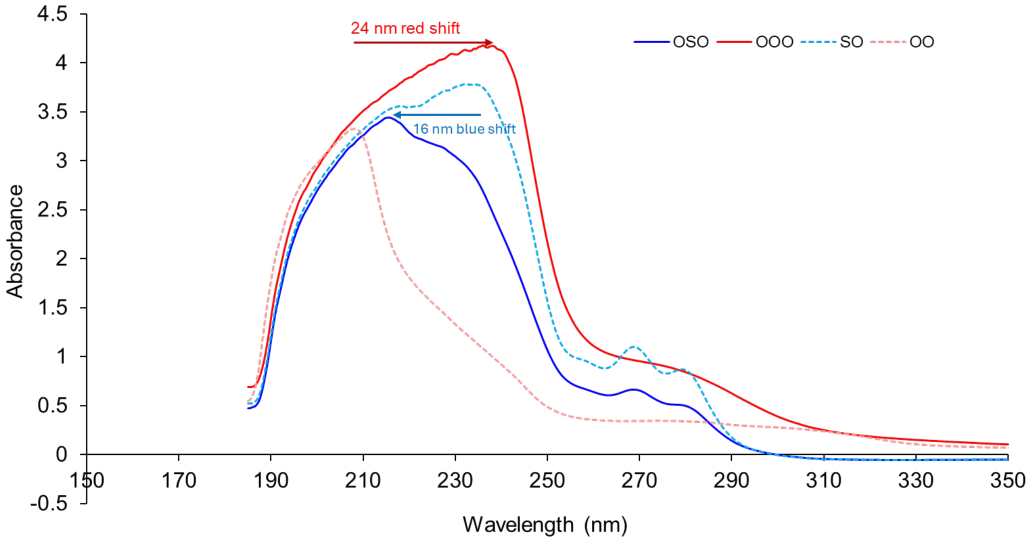

Supplementary Figure S1. UV spectrum (scan range 190–350 nm) of the sunflower oil (SO), ozonated sunflower oil (OSO), olive oil (OO) and ozonated olive oil (OO).

Supplementary Figure S2

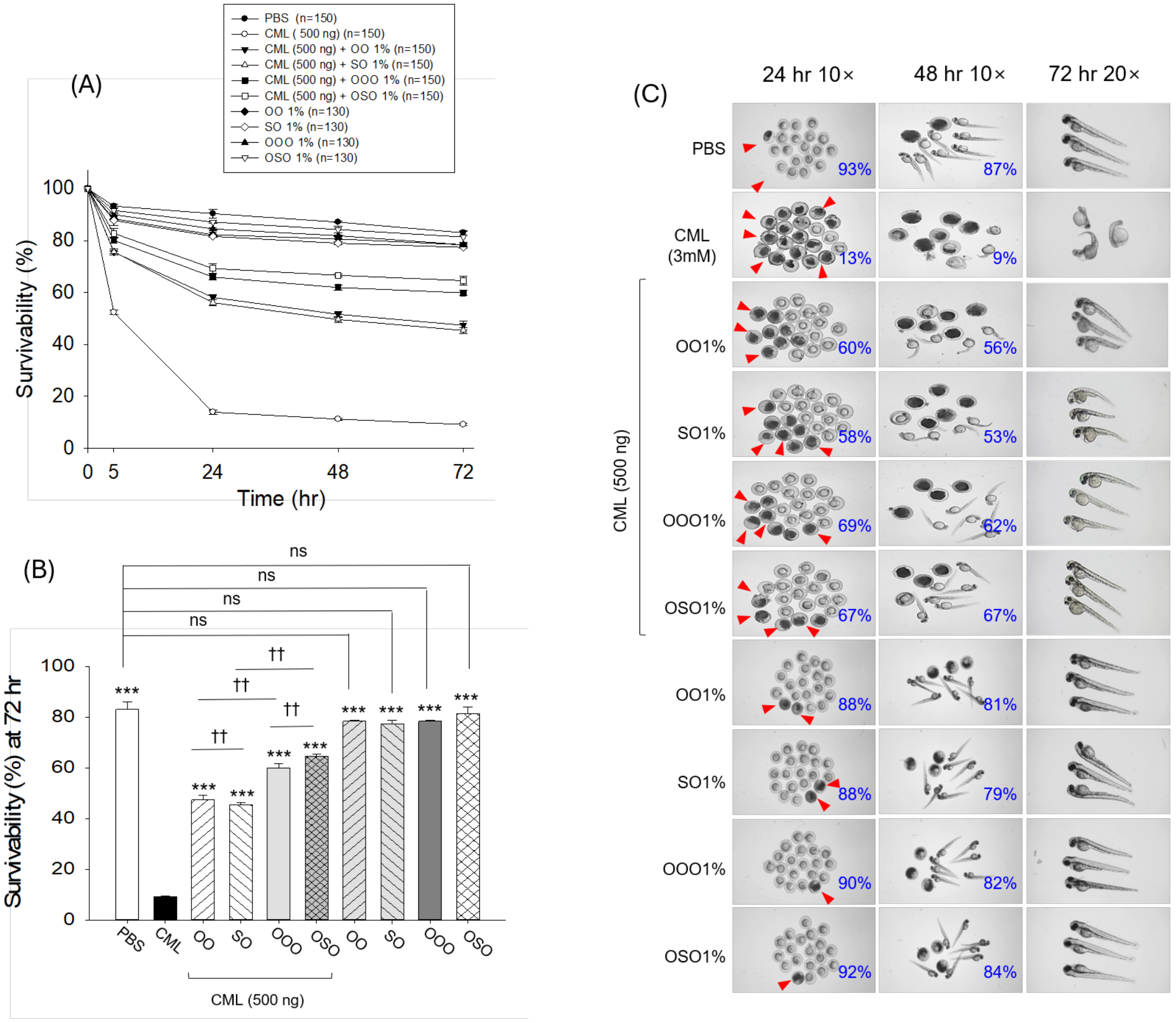

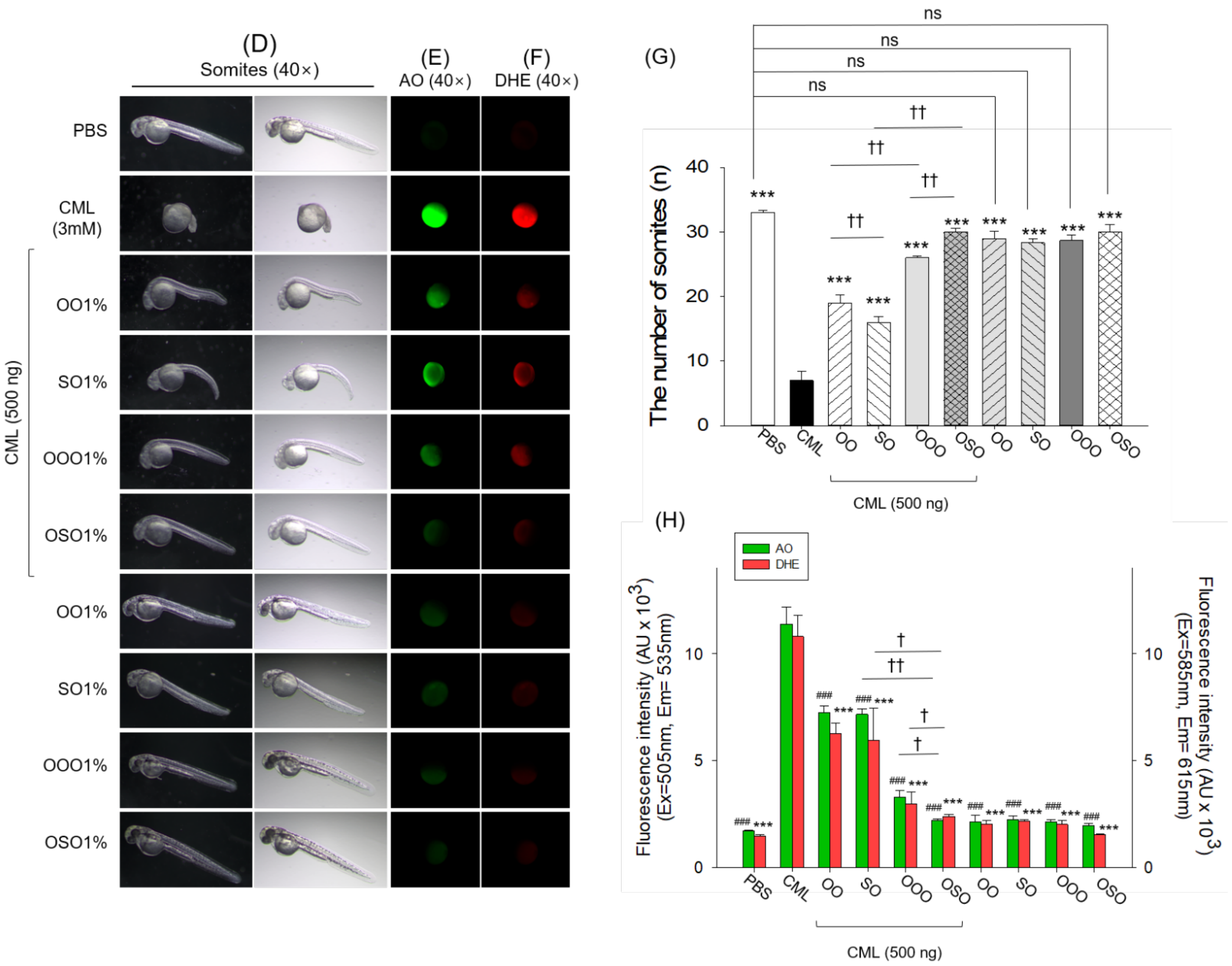

Supplementary Figure S2: A comparative effect of olive oil (OO), sunflower oil (SO), ozonated olive oil (OOO), and ozonated sunflower oil (OSO) on the carboxymethyllysine (CML) induced toxicity in zebrafish embryos. (A) Survivability kinetics (0-72 hr). Each data point represents the mean ± SEM obtained from 150 embryos divided into three groups (n = 3, 50 × 3 = 150). (B) Embryos survivability at 72 hr post treatment. (C) Images of embryos post 24, 48, and 72 hr treatment. The red arrow indicates dead embryos. (D) Representative images depicting somites. (E) and (F) dihydroethidium (DHE) and acridine orange (AO) staining, respectively. (G) and (H) Quantification of average somite counts and fluorescent intensity, respectively. The statistical difference ($p < 0.001$; ***) was determined using one-way ANOVA with Tukey's post hoc analysis for the CML-injected group. The $p <$ 0.05 (†) and $p < 0.01$ (††) represent the statistical difference between the marked groups determined by *t*-test. A non-significant difference is depicted by "ns".

Supplementary Figure S3

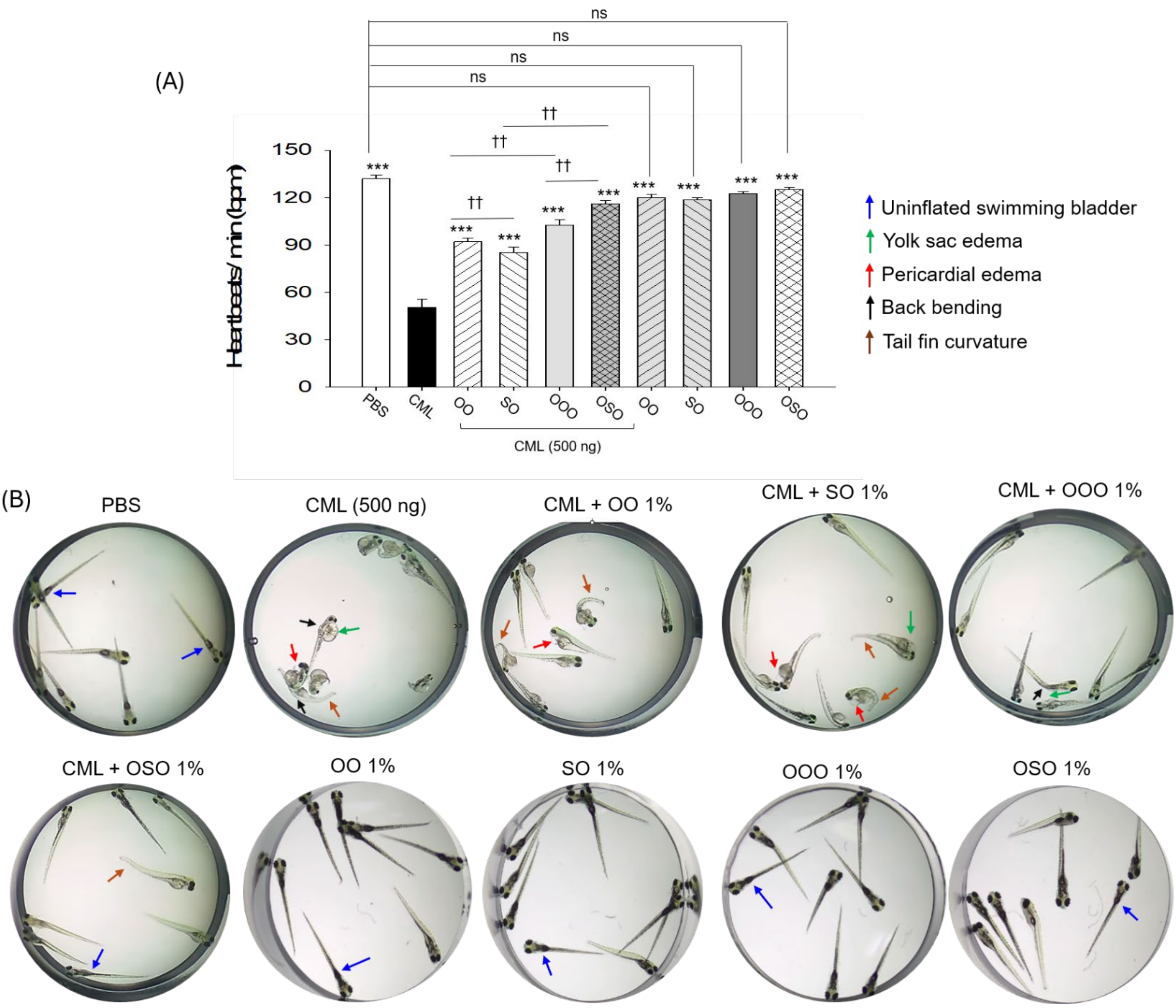

Supplementary Figure S3: The comparative effect of olive oil (OO), sunflower oil (SO), ozonated olive oil (OOO), and ozonated sunflower oil (OSO) on the CML-triggered adverse events in zebrafish embryos. (A) Heartbeat calculated at 72 hr post treatment. Each point represents the mean ± SEM from three (n = 5) independent experiments. (B) Pictorial view of the representative embryos at 144 hr post-treatment. Blue arrows highlight the uninflated swimming bladder, green and red arrows represent yolk sac edema and pericardial edema, respectively, while brown and black arrows represent tail fin curvature and back bending, respectively. The statistical difference $p < 0.001$ (***) corresponds to the CML-injected group using one-way ANOVA following Tukey's post hoc analysis. The $p < 0.05$ (†) and $p < 0.01$ (††) represent the statistical difference between the marked groups determined by the *t*-test.
